# A Delayed and Rather Unusual Presentation of a Bladder Injury after Pelvic Trauma: 5 Years after a Road Traffic Accident

**DOI:** 10.1155/2014/873079

**Published:** 2014-03-04

**Authors:** Nikolaos Davarinos, John Thornhill, JP McElwain, David Moore

**Affiliations:** Department of Trauma & Orthopaedics, Tallaght Hospital, Dublin 24, Ireland

## Abstract

Associated injuries frequently occur in patients who sustain fractures of the pelvis. Specifically, high-energy trauma resulting in pelvic fractures places the bladder and urethra at risk for injury, often resulting in significant complications. Timely identification and management of genitourinary injuries minimize associated morbidity. Prompt injury identification depends upon a systematic evaluation with careful consideration of the mechanism of injury. Physical examination is pertinent as well as analysis of the urine and appropriate diagnostic imaging. Despite such increased vigilance genitourinary injuries get missed and delayed presentations in the order of a few weeks have been well documented. To our knowledge, this is the first report of its kind in the literature showing such a particularly delayed (5 years) and rather unusual presentation of a bladder injury after pelvic trauma.

## 1. Introduction

Pelvic fractures are usually the result of high-energy trauma and may have associated soft tissue and organ damage resulting in significant morbidity and mortality in these patients. The typical profile of such patient depicts a young male individual in his 30s involved in a high-energy road traffic accident (RTA) [1].

There may be multiple-system involvement following injury. Injuries to the lower genitourinary (GU) tract alone are not life threatening, but their association with other potentially more significant injuries necessitates an organized approach to diagnosis and management. Other injuries often take priority over injuries to the GU system and may initially interfere or postpone a complete urologic assessment. Coordinated efforts between various services caring for the patient are crucial to ensure comprehensive care [2, 3].

Initial evaluation of the injured patient should follow the protocols of the Advanced Trauma Life Support program of the American College of Surgeons [4].

The lower GU tract comprises the urinary bladder, urethra, and external genitalia. Most bladder injuries occur in association with blunt trauma. Eighty-five percent of these injuries occur with pelvic fractures, with the remaining 15% occurring with penetrating trauma and blunt mechanism not associated with a pelvic fracture (i.e., full bladder blowout) [5].

Bladder injuries are best classified as intraperitoneal and extraperitoneal. Extraperitoneal bladder injuries account for 65–85% of bladder injuries and are usually associated with pelvic fractures, especially pubic ramus fractures (95%). Intraperitoneal bladder injuries account for 15–35% of bladder injuries and are infrequently associated with pelvic fractures. These injuries may be due to blunt rupture of a distended bladder or penetrating injury [5].

In this paper, we are reporting such a case of bladder injury in a young female after pelvic trauma due to an RTA with a delayed and rather unusual presentation.

## 2. Case Report

A 30-year-old female was involved in an RTA 5 years prior to her presentation to our unit. She was diagnosed with an isolated right superior pubic ramus fracture at the time. Conservative treatment and physiotherapy lead to an uneventful recovery overall.

She had unresolved residual discomfort around the right groin area intermittently. Five years after the accident, she was still experiencing right groin discomfort especially during exercise and she periodically had microscopic haematuria. This intermittent, exercise-induced discomfort with associated haematuria was treated with short courses of antibiotics for presumed urinary tract infections. These courses of antibiotic therapy were unsuccessful and eventually prompted GP specialist referral.

**Figure 1:**
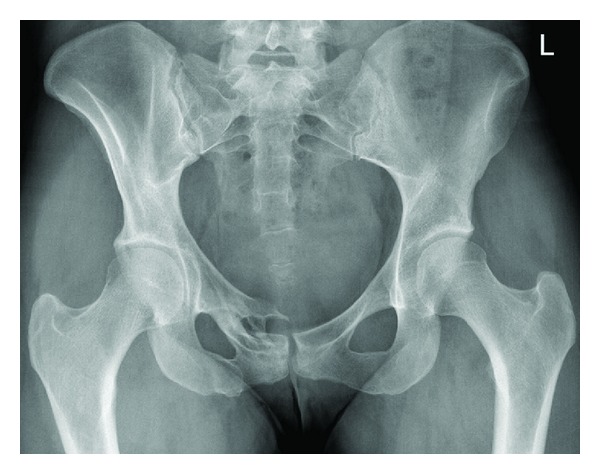
Anteroposterior radiographs of the pelvis demonstrating the right superior pubic fracture 5 years after the RTA.

New investigations were carried out. Plain film radiographs revealed a malunited right superior pubic ramus fracture (Figure 1).

**Figure 2:**
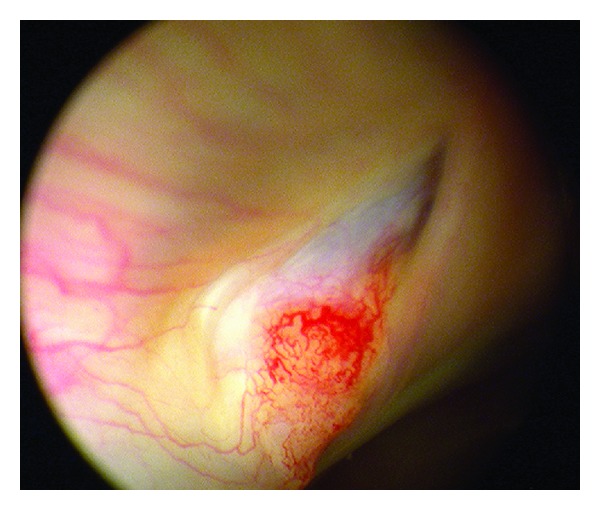
Intraoperative photographs from cystoscopy demonstrating a spike of bone in the bladder.

A subsequent cystoscopy revealed the image shown in Figure 2.

**Figure 3:**
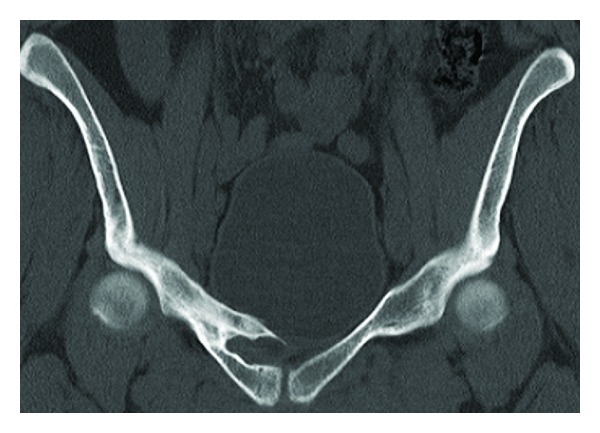
CT coronal slice of the pelvis demonstrating a malunited right superior pubic fracture with a bony spur projecting through the bladder wall.

**Figure 4:**
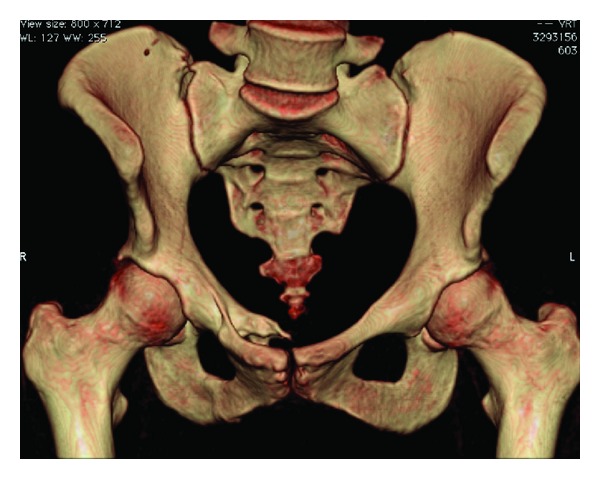
3D reconstruction of pelvis depicting the right pubic ramus injury from the RTA 5 years prior.

More investigations including CT and MRI were obtained to provide better imaging (Figures 3 and 4).

**Figure 5:**
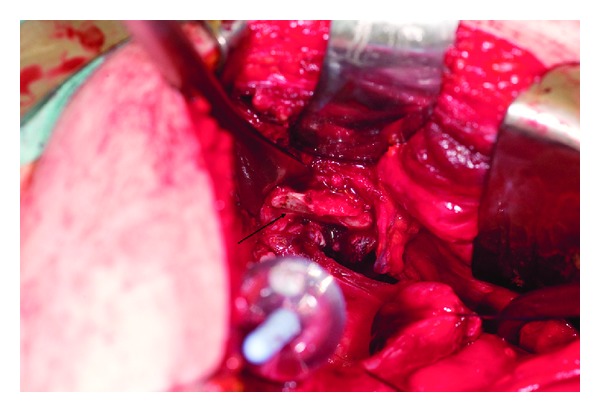
Open bladder with visible bone spur (black arrow).

**Figure 6:**
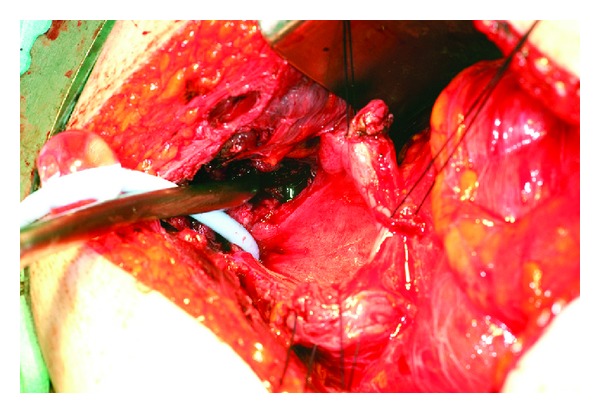
Open bladder after bone spur removal.

The patient was admitted to our hospital. After orthopaedic and urology investigations and consultations, she had an elective excision of the bony spur from her right superior pubic ramus in conjunction with bladder repair. An intraperitoneal approach through a vertical lower abdominal midline incision was chosen. The main intraoperative findings were as follows. The bladder was densely adherent anteriorly and the right side was mobilised and the bladder was opened in the midline. The bone spur was evident at this point (Figure 5) and removed with an osteotome (Figure 6). There was no involvement of the right ureter. Finally the bladder was irrigated and closure was performed.

The patient made an uneventful postoperative recovery.

## 3. Discussion

In the setting of an acute injury, evidence based recommendations mandate a cystogram if there is haematuria and concomitant pelvic fracture. Yet delays (usually hours to days) and difficulties in the diagnosis of lower urologic injuries in the context of pelvic fractures have been documented in the literature [6, 7].

This is, to our knowledge, the first case in the literature with such a long delay in diagnosis: 5 years after the injury. Prompt recognition and early management of these urological injuries are necessary in order to reduce morbidity and mortality. Difficulties arise most often in the multitrauma patients when lifesaving measures or damage-control surgery may delay the diagnosis and treatment of lower urinary tract injuries [8].

With the increased concentration of management of injured patients in trauma centers, both orthopaedic surgeons and urologists are recognising increasing numbers of lower urinary tract injuries [8].

Our case report presents an unusually delayed presentation of bladder injury after significant pelvic trauma. It highlights the importance of a good history and examination especially in the presence of persistent and unexplained symptoms. It also supports the view that a high volume of cases, standardized trauma protocols, and high index of suspicion can be proved to be of great benefit and help avoid delays in diagnosis and prompt management of such injuries.

Although it is probably safe to assume that bladder injuries will continue to be a rare event, they will occur. It has been shown that a high index of suspicion and rapid utilization of all resources is our only way of diagnosing these injuries in a timely manner. Bony spicules in close proximity to the bladder wall have been speculated to be an increased risk for bladder rupture if seen and should be carefully examined [7].

## 4. Conclusion

Lower urinary tract trauma can lead to significant morbidity when diagnosed late; urologists may only encounter a handful of these injuries in their career. When evaluating patients with blunt (RTA) or penetrating (gunshot wounds or stabbings) lower abdominal trauma doctors must have a high index of suspicion for urological injury, especially bladder and urethral injuries. Most importantly, persistent yet unexplained genitourinary symptoms with a history of pelvic injury in association with a significant, high-energy mechanism (i.e., RTA) in the past should prompt full urological history and examination.
